# Obstacles to the coordination of delivering integrated prenatal HIV, syphilis and hepatitis B testing services in Guangdong: using a needs assessment approach

**DOI:** 10.1186/s12913-015-0760-0

**Published:** 2015-03-24

**Authors:** Jianhong Xia, Shannon Rutherford, Yuanzhu Ma, Li Wu, Shuang Gao, Tingting Chen, Xiao Lu, Xiaozhuang Zhang, Cordia Chu

**Affiliations:** Centre for Environment and Population Health, Nathan campus, Griffith University, 170 Kessels Road, Nathan, Brisbane, QLD 4111 Australia; Guangdong Women and Children Health Care Hospital, 521-523 Xingnan Road, Panyu district, Guangzhou, Guangdong 511442 China

**Keywords:** Integrated prenatal testing services, Prevention of mother-to-child transmission of HIV

## Abstract

**Background:**

Integration of services for Prevention of Mother-To-Child Transmission of HIV (PMTCT) into routine maternal and child health care is promoted as a priority strategy by the WHO to facilitate the implementation of PMTCT. Integration of services emphasizes inter-sectoral coordination in the health systems to provide convenient services for clients. China has been integrating prenatal HIV, syphilis and hepatitis B testing services since 2009. However, as the individual health systems are complex, effective coordination among different health agencies is challenging. Few studies have examined the factors that affect the coordination of such complex systems. The aim of this study is to assess the effectiveness of and examine challenges for integrated service delivery. Findings will provide the basis for strategy development to enhance the effective delivery of integrated services.

**Methods:**

The research was conducted in Guangdong province in 2013 using a needs assessment approach that includes qualitative and quantitative methods. Quantitative data was collected through a survey and from routine monitoring for PMTCT and qualitative data was collected through stakeholder interviews.

**Results:**

Routine monitoring data used to assess key indicators of coordination suggested numerous coordination problems. The rates of prenatal HIV (95%), syphilis (47%) and hepatitis B (47%) test were inconsistent. An average of only 20% of the HIV positive mothers was referred in the health systems. There were no regular meetings among different health agencies and the clients indicated complicated service processes. The major obstacles to the coordination of delivering these integrated services are lack of service resource integration; and lack of a mechanism for coordination of the health systems, with no uniform guidelines, clear roles or consistent evaluation.

**Conclusions:**

The key obstacles that have been identified in this study hinder the coordination of the delivery of integrated services. Our recommendations include: 1) Facilitate integration of the funding and information systems by fully combining the service resources of different health agencies into one unit; 2) Establish regular meetings to facilitate exchange of information and address problems; 3) Establish a client referral network between different health agencies with agreed guidelines, clear roles and consistent evaluation.

## Background

The fight to control HIV/AIDS has entered its fourth decade. However, in 2012, there were still about 260,000 newly infected HIV cases in the world, and approximately 90% of childhood cases were infected by mother-to-child transmission (MTCT) [1]. In China, in 2012, there were 782 children reported to be HIV infected by MTCT [2]. These figures highlight the remaining challenge to achieve elimination of HIV infection in children [3].

In some developed countries, zero HIV infection in children has been achieved because of effective implementation of the comprehensive measures for prevention of MTCT of HIV/AIDS (PMTCT) [1]. However, in China, the difficulties in coordination among health service systems along with various access issues have hampered the successful implementation of interventions for PMTCT.

A priority strategy to reduce MTCT of HIV promoted by the WHO [4,5] is the integration of services for prevention of MTCT into routine maternal and child health care and other health services. Integration of services means health care that is seamless, smooth and easy to navigate for the users, and for the providers, it means that separate technical services, and their management support systems, are provided and managed in a closely coordinated way [6]. The characteristics of integrated services are related to linkage, co-operation, sharing and being client focused. Available evidence suggests that an integrated approach will help optimize health outcomes, not only for PMTCT, but also by making the best use of resources for all aspects of maternal, newborn and child health [1,4,5]. However, delivery of integrated services requires a shift in the management model from providing stand-alone PMTCT services to integrating services and such a shift will require more planned management within a more complex system [7-13]. In some countries, models have been implemented to improve the delivery of integrated PMTCT service. One such model tries to improve effectiveness of service delivery by providing one-stop-services or point-one-care, along with decentralization and integration of service procedures and technologies into Primary Health Care (PHC) services [14,15]. Another model utilizes community lay workers to shift tasks from overworked health workers to address human resource shortages [16,17]. However, these models don’t examine how to facilitate coordination among complex health systems to deliver integrated services effectively because their service systems are not separate.

China initiated the process of integrating the PMTCT service with the antenatal care service in 2003 [18]. Since 2009 this has been extended to include prevention of MTCT of syphilis and hepatitis B [18]. Prenatal HIV, Syphilis and Hepatitis B Testing (PHSHT) services are an important part of PMTCT services, helping to identify positive pregnant women for further intervention. In China, integrated PHSHT services, including pre-test counselling, an initial screening test, confirmation test, post-test counselling and referral services, are not provided by one health agency. Four different health agencies –Maternal and Children Health care (MCH) hospitals, the Centre for Disease Control (CDC), Infectious Disease Treatment (IDT) hospitals and the Clinic for Prevention of Sexual Transmission Disease (CPSTD)– must work together to provide these integrated PHSHT services. In addition, the vertical nature of government in China means that there are five tiers in every health agency from the national to township level. In this complex health service system, the task of integration is highly problematic. Among the different health agencies, there are different professional backgrounds and operational procedures, and different capacities for delivering services. These difficulties lead to higher requirements for coordination. However, few studies have identified the factors that affect coordination in such complex systems. This study attempts to fill this gap by assessing the effectiveness of, and examining challenges to, integrated service delivery.

This paper will first provide a quantitative assessment of coordination in the delivery of integrated PHSHT services in China. Then, a qualitative assessment is used to examine in-depth the reasons behind these statistics, and identify the obstacles to coordination of health systems in the delivery of these integrated services. The findings provide the basis for strategy development to improve the coordination and enhance the delivery of integrated services.

## Methods

We conducted this study between June and December 2013 in Guangdong province. Guangdong has the sixth highest accumulative HIV/AIDS cases in 2012 in China [2]. The structure of administration in the Chinese health service system is similar in all 31 provinces. The PMTCT pilot project has been operating since 2003. Consistent with other provinces, Guangdong’s PMTCT health service system includes both the vertical and horizontal systems. Vertically there is the provincial health system, 21 city level health service systems, 110 county level health service systems and more than 2000 township hospitals. There are four types of institutes related to delivering integrated PHSHT services. They include the MCH hospitals, the CDC system, the IDT system and the CPSTD system. There is a lot of experience in the PMTCT in Guangdong province, though similar challenges exist in the whole country.

We utilized a mix of qualitative and quantitative methods with a needs assessment framework to evaluate effectiveness of and examine challenges for, coordination in the delivery of integrated PHSHT services. A needs assessment framework involves a process of assessing needs, including issues of concern, health determinants and solutions, capacity, and resources to inform the direction, scope and conduct of community health promotion [19,20]. It can be viewed as a tool to identify what a particular stakeholder needs. The needs assessment framework has made a good use of advantages of quantitative and qualitative methods in this study. The study conducted quantitative CAN to understand the extent of the issues in integrated PHSHT services delivery, and used qualitative CNA to examine the reasons behind the issues and identify barriers to integrated PHSHT services delivery.

**Figure 1:**
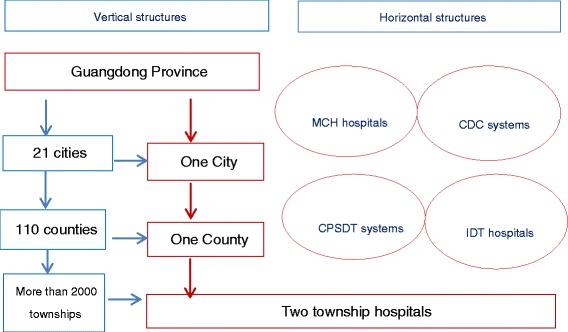
**The structure of research sites.**

In this study, we selected all city and county MCH hospitals and all township hospitals in Guangdong province to conduct the quantitative survey of the service providers (health agencies) by the routine monitoring for PMTCT program. Considering the characteristic of sample selection for qualitative research, and the resources and time limitations of this research study, we only selected all the provincial institutes related to the PMTCT service, all the health agencies of one city, and of one county as well as two township hospitals in which to conduct the qualitative needs assessment and the survey of the service users (pregnant women). The selected city which is one of 21 cities in Guangdong has the highest report of HIV/AIDS cases. Many programmes for prevention of HIV are piloted in the selected city, so this city has greater experience but also greater needs for integrated PMTCT services. The selected city includes four counties and seventy eight townships. There are a similar management structure in every city, county and township, and all cities, counties and townships carry out same policies and measures for PMTCT. The structure of research sites is shown in Figure 1.

### Quantitative data collection and analysis

In this study, quantitative data were collected from a questionnaire survey and routine monitoring undertaken for the PMTC program. The purpose of the survey was to understand service users’ views on integrated PHSHT services. Based on the concepts of integration of health services [6] and relative literature on demands of pregnant women and its influencing factors in PMTCT [21-23], the key categories of measures in the survey included pregnant women’s knowledge of PHSHT services and their satisfaction with the services (See Table 1). Several key informants were informally involved in the fieldwork preparation to help contact pregnant women and to conduct informed consent among the pregnant women. The sample size of each level from province, city, county to township was calculated as follows n = Z^2^_α/2_ (1-P)/ε^2^P, (Z_α/2_ = 1.96, which stands for critical value of standard normal distribution, α(size of a test) = 0.05; ε = 0.2, which stands for the relative error; P = 0.23%, which stands for the average incidence of HIV in pregnant women). An average of 400 pregnant women was included at each level hospital. Thus, a total sample of 1600 pregnant women was selected randomly from the outpatient of one province, one city and one county MCH hospitals and two township hospitals. The survey data of the health agencies from routine monitoring for the PMTCT program, which was established in all city and county MCH hospitals (n = 132) and all township hospitals (n = 1317) in 2011, were used to measure the effectiveness of the PMTCT program in Guangdong. Based on the concepts and relevant literature of integration of health services [6,12,13], the key categories of measures of coordination for integrated services delivery included consistency in the test services, referrals and regularity of communication among the key health agencies (See Table 1).

**Table 1:** **Measures of effectiveness of integrated PHSHT service delivery**

**Service users’ views**		**Measures of coordination among service providers**
**Knowledge of PHSHT services**	**Satisfaction with the services**	
Whether the users(pregnant women)	Whether the users (pregnant women) are satisfied with the services	-Consistency of the test services
		Whether the rates of prenatal HIV, syphilis and hepatitis B test are consistent
-know about HIV, syphilis and hepatitis B		
-know about MTCT of HIV, syphilis and hepatitis B		-Referrals among the key health agencies
		Referral rates of HIV positive pregnant women
		-Regularity of communication among the key health agencies
		Frequency of regular meetings among the health
-know that MTCT of HIV and syphilis can be prevented		
-understand the HIV and syphilis test		
-know that HIV, syphilis and hepatitis B tests are free		
-know when is the best period to undergo test during pregnancy		
-receive test results		
	The reasons for dissatisfaction with the services	

All the quantitative date was entered using Epidata software, and analysed using SPSS 17.0. Descriptive statistics were used to assess the effectiveness of coordination among different health agencies in the delivery of integrated PHSHT services.

### Qualitative data collection and analysis

For this study, qualitative data were collected from in-depth individual interviews and focus group interviews. The purpose of the qualitative investigation was to identify the reasons behind statistics collected and to examine the factors that affect coordination in the delivery of integrated PHSHT services. The interviewees for interviews included the PMTCT program managers/administrators and experts from the province (n = 8), city (n = 4) to county (n = 4), as well as HIV positive pregnant women (n = 6). There were 4 focus group interviews with health workers from provincial, city, county and township health agencies. Several key informants were informally involved in the fieldwork preparation to advise the researcher about the field site and to help contact potential interviewees. They also worked as local intermediaries for the researcher to reach HIV positive pregnant women. The interviews with HIV positive pregnant women focused on satisfaction with the services and their needs related to the services; and the interviews for the managers and health staff focused on perspectives, needs and challenges for coordination among different health agencies in delivering integrated PMTCT services. All interviews were recorded and notes were taken during the conversations.

The qualitative data analysis utilized manual thematic analysis. It involved three steps: defining issues and factors, forming themes and categories among factors, and building an explanatory framework to develop an in-depth understanding of challenges for coordination among these complex service systems in the delivery of integrated PMTCT services. In this study, two themes of challenges for integrated PMTCT services delivery were created, including the poorly integrated service resources (human resources, financial support and information systems) and a lack of a mechanism for coordinated management (lack of the referral and communication networks with agreed guidelines and clear agency roles and inconsistent outcome evaluation).

An ethical review of this study was submitted to the Human Research Ethics Committee in Griffith University before beginning the fieldwork, and ethical clearance was obtained from that committee (ENV/71/12/HREC). Local Chinese approval of the study protocol was also obtained from the Eighth People’s Hospital, Maternal and Children Health Hospital, Institute of HIV Prevention and Control, and Administration Department of Guangdong Health Bureau, which presented approving for access to City, County and Township related hospitals. All the written informed consent for participation was obtained from participants, including service users (1600 pregnant women, six HIV positive pregnant women) and service providers (managers, administrators, experts and health workers from different health agencies).

## Results

This section will present outcomes of the needs assessment including the effectiveness of, and obstacles to the coordination of the integrated PHSHT service delivery in China. The participants were service users (pregnant women, including six HIV positive pregnant women) and service providers (managers, administrators, experts and health workers from different health agencies).

### The effectiveness of integrated PHSHT service delivery

The effectiveness of the service delivery was evaluated using quantitative methods. The indicators related to the coordination of the service delivery and included clients’ views, consistency of the test service, referrals and regularity of communication among the key health agencies.

#### Clients ‘views

In this study, clients were the pregnant women who regularly attended prenatal care. Their views reflected their needs and service effectiveness. Two key categories of measures including knowledge of the PHSHT services and satisfaction with the services described their views. Table 2 presents pregnant women’s views on integrated PHSHT service delivery. In the first section on knowledge of the PHSHT services, the results reflected that the pregnant women have not enough knowledge about the PHSHT services. In the second section on satisfaction with the services, the results indicated that women found the service processes were too complicated and took a long time.

**Table 2:** **The pregnant women’s views on integrated PHSHT services (n = 1600)**

**Indicators**	**The pregnant women’s views**
Knowledge of the services	--10% of pregnant women had not heard of HIV
	--34% of pregnant women were unaware of MTCT of HIV, syphilis and hepatitis B
	--59.2% of pregnant women did not know MTCT can be prevented
	--43% of pregnant women did not know whether they had undergone HIV and syphilis test or not
	--39% of pregnant women did not know the optimal time for HIV and syphilis tests during pregnancy
	--52% of pregnant women did not know their test results
Satisfaction with the services	--30% of pregnant women were unsatisfied with the test services
	The reasons include:
	--73.9% of pregnant women thought it took a long time
	--37% of pregnant women thought the test services processes were too complicated

#### Consistency of the test services

Statistics on numbers of test services are used in this study to provide an assessment of the integration of perinatal HIV, syphilis and hepatitis B testing services. Testing is crucial to help identify positive pregnant women for further intervention in order to reduce perinatal HIV, syphilis and hepatitis B transmission. The rates of testing are the key indicators for evaluating the effectiveness of the prenatal test services. The national standard for rates of HIV, syphilis and hepatitis B test is a rate that is higher than 85%. In this study, the average rates of HIV tests at a provincial and city level were both above the national standard, but the syphilis and hepatitis B test rates did not reach the national standards. In addition, the rates of HIV, syphilis and hepatitis B test were inconsistent, despite the fact that the three testing services have been integrated. The results are described in Table 3.

**Table 3:** **The prenatal testing rates of HIV, syphilis and hepatitis B between January and October in 2013**

**The prenatal testing rates**	**Province level**	**City level**
HIV	91%	95%
Syphilis	77%	47%
Hepatitis B	79%	47%

#### Referrals among the key health agencies

The process of confirming HIV infection tests requires the client to navigate three different level hospitals in the investigation area. The township hospital only has the ability to conduct rapid HIV screening. If the HIV rapid test result is positive, the client needs to go to the county CDC to undergo the HIV initial screening test. If this test result is still positive, the client needs to go to the city CDC to undergo an HIV confirmation test. If there is a lack of a referral network between laboratories at different levels, this would, in turn, create obstacles to the successful delivery of the HIV confirmation test. In this study, HIV confirmation test results took an average of one month to get because of the lack of coordinated referrals among different level laboratories. The process should take just one week. Additionally, an average of only 20% of the HIV positive mothers were referred to hospitals for continuous monitoring and treatment services. The results indicate significant issues of coordination in the referral network.

#### Regularity of communication among the key health agencies

Communication is important for different health agencies to facilitate information exchange and to address problems in the delivery of integrated services. In this study area, there are no regular meetings among the four key health agencies involved in the delivery of these integrated services. This highlights a significant challenge for the coordinated management of the delivery of integrated services.

### Obstacles to the coordination of integrated PHSHT service delivery

A number of obstacles to the coordination of integrated PHSHT service delivery were identified from the interviews of users and providers. These challenges related to the poorly integrated service resources and a lack of a mechanism for coordinated management, which both negatively impact on the effective delivery of integrated PHSHT services in China.

#### The poorly integrated service resources

Service resources represent the resources available to service providers for the delivery of these integrated services, and hence are an important element of service capacities. The key service resources include human resources, financial support and information systems. Interviewees reflected that these service resources have not been integrated for the delivery of the integrated services.

##### Human resources

In terms of the human resources, some of the comments from a MCH hospital were:

*“Only I and another doctor were appointed to attend national training about PMTCT of HIV in my hospital. Because of the heavy workload, other doctors have no chance to attend the training, so we two take on to look after all HIV positive pregnant women in my hospital.”*

*“For syphilis positive pregnant women, I really do not know how to deal with them because I have never received the relevant training; I have to ask them to go to the STD clinic.”*

Other comments from CDC systems and CPSTD systems were:

*“Our clinics rarely provide test services for pregnant women; usually we suggest they go to MCH hospitals”*

These comments suggest that there is not enough staff trained in MCH systems to look after patients with HIV, or syphilis, or hepatitis B, and there is a lack of uniform test counselling technology guidance and training for providing these integrated services. A similar concern has been identified in CDC systems and CPSTD systems, suggesting that the lack of staff with multiple skills has a negative effect on quality of integrated services.

##### Financial support

Some participants expressed concern about the impact of inadequate funding and fund allocation for the effective delivery of these integrated services. The following comments were made by the managers of MCH hospitals and CDC systems:

*“For free PHSHT services, the government only provides free test reagents to our hospital, no funds to support lab infrastructure, quality management and human resources for the lab. There is no motivation for us to implement free test services”*

*“It is not easy for us to allocate government funds to MCH systems for PMTCT of HIV, because there are many overlaps between us, and there is no clear responsibility for us in PMTCT of HIV.”*

The comment above suggests that funds used for PMTCT of HIV is not adequate, and financial support for interventions of PMTCT still depends on special project fund allocation. In utilization of funds there is no cooperation between MCH systems and CDC systems to realize sharing of funds.

##### Information systems

Iinformation systems that relate to information sharing and communication are also an important service resource in building cooperation between different health agencies for the effective delivery of integrated services. However, there is the lack of integration of the information systems between different health agencies. The information for PMTCT is provided only by the MCH system. Some respondents from MCH hospitals expressed concerns about information sharing and communication in the delivery of the services. One respondent from the MCH system commented:

*“We have an information system for surveillance of PMTCT of HIV. I know that the CDC has an information system for surveillance of PHIV, but there is no channel to help us communicate the information to each other.”*

*“I do feel I am doing lots of overlap work in information reporting. There are two information systems for surveillance of HIV and so much cross information in the two systems. We cannot understand why the two information systems cannot be integrated into one information system.”*

The statements above suggest that there is a lack of integration of information between the MCH and CDC systems. Information integration is significant for facilitating information sharing and communication for the effective delivery of integrated services.

#### Lack of a mechanism for coordinated management

Effective integration of health services depends on effective coordination of the relevant health systems. However, the assessment of the stakeholders’ needs reflected that there was a lack of a mechanism for coordinated management in the delivery of integrated PHSHT services.

##### Lack of the referral and communication networks with agreed guidelines and clear agency roles

The current study found that referrals and communication, management guidance, roles and responsibilities, are all unclear, confusing and duplicated. These issues are reflected in the comments of health staff in both the MCH and CDC systems. Typical comments are:

*“We provide test services, but test reagents supply and the lab qualification and quality management are managed by different health agencies. HIV test relevant services are managed by CDC systems, and syphilis and hepatitis B test relevant services are managed by Center for Prevention of Sexually Transmitted Diseases or Centers for Clinical Testing. It is very hard for us to coordinate test reagents supply, quality control of tests and test referrals, particularly for the pregnant women with positive test results. There is no uniform PHSHT services management guideline.”*

These comments suggest that the current policies of integrated services do not offer concrete guidance to help achieve coordinated management in the delivery of these integrated services among key health agencies. A number of issues were raised by the managers in MCH systems in regard to coordination in test reagents supply and lab management.

In addition, some comments reflected that roles and responsibilities of different health agencies were unclear or confusing, and referral and communication networks were not established in delivering integrated services. A comment from one of the managers of CDC systems was more specific:

*“We have been providing stand-alone PHIV services. Mother-to-child transmission of HIV, syphilis and hepatitis B relates to pregnant women. Maternal and children’ health hospital should take on all work for PMTCT of HIV.”*

##### Inconsistent outcome evaluation

Furthermore, a number of issues were raised by staff in regard to evaluation of the outcomes of the delivery of integrated services. The staff from MCH systems reflected that the effectiveness of delivery of these integrated services is evaluated only in MCH systems rather than in all health agencies relevant to these integrated services.

*“It’s unfair that the effectiveness of PMTCT is only used to evaluate the services of MCH systems rather than those of CDC systems or CPSTD systems.”*

However, the managers from CDC and CPSTD indicated:

*“All effectiveness indices of PMTCT are related to MCH systems; and we do not pay attention to these indices because they are not so relevant to our work.”*

These comments suggest that outcome evaluation systems were not integrated among the different health agencies. Some agencies were thoroughly evaluated, others were not. This situation indicates the problem of coordination between MCH systems and CDC and CPSTD systems; because the inconsistent evaluation of outcomes means that CDC and CPSTD systems take no responsibility for the effectiveness of these integrated services.

## Discussion and recommendations

Integration of PHIV services within antenatal care is recommended by the WHO as a priority strategy to facilitate the implementation of PMTCT. In some contexts, there is evidence of the effectiveness of this integrated approach [1,4,8-11,15-17]. However, integration of health services is interpreted and applied in various ways in different health systems, particularly in the delivery of integrating multi-services in more complex systems [7,13]. In China, since the health systems are complex, effective coordination among different health agencies is challenging. There is a lack of research that systematically examines the factors that affect coordination among a complex health system for the delivery of integrated PMTCT services. This study was designed to determine the effectiveness of, and obstacles to coordination of integrated PHSHT service delivery in China.

The current study found that the coordination of delivering integrated PHSHT services was problematic. The rates of HIV, syphilis and hepatitis B prenatal test were inconsistent; and about 80% of HIV positive pregnant women were not referred to IDH hospitals for continuous monitoring and treatment services. Additionally, there was a lack of regular meetings among the four key health agencies; and the pregnant women thought the service processes were too complicated and time-consuming. The results are consistent with relevant literature on assessment of the service providers and users in the delivery of integrated PMTCT services [12,13,21-23].

The possible reasons behind these results also have been identified in this study. There were two categories of key factors, including the poorly integrated health resources and a lack of a mechanism for coordinated management among the health systems, which created the obstacles to coordination of integrated PHSHT service delivery in China. This study found that failure to coordinate human resources, funding and information systems across key agencies has hampered the delivery of integrated PHSHT services. These findings are consistent with those of previous studies that have examined the effect of inadequate service resources or weak health systems on the effective delivery of integrated services [12,14,24-28]. Another finding was a lack of a mechanism for coordinated management among the complex health systems. The complex service processes with no agreed uniform guidelines, clear roles and consistent outcome evaluation may bring more difficulties to establish the referral and communication networks; in turn, a lack of the referral and communication networks or a lack of uniform guidelines, clear roles and consistent outcome evaluation systems also may lead to more complicated service processes. All these factors collectively contribute to impede effective coordination of an integrated service delivery system. The previous studies identified the challenges for the management of integrated services including weak global governance, unclear definition and different perspectives [12,13,27,29]. However, these earlier findings were examined in the delivery of integrated services for sexual and reproductive health and HIV or other integrated services, and did not systematically identify the key obstacles to the coordination within and among the complex health service contexts. The findings of this study provide a more comprehensive understanding of the obstacles to the coordination of integrated PHSHT service delivery.

Based on the challenges identified from this study and the previous studies, this study also provide the foundation for strategy development to improve the coordination for the effective delivery of integrated PHSHT services in China. As for addressing human resource shortage and promoting integration of health resources (funding, infrastructure, procurement and supply and information systems), strengthening primary health service and decentralization of services for simplifying the service processes, facilitating task-shifting and community participation are all key factors for successful delivery of integrated health services. we recommend to facilitate integration of the funding and information systems by fully combining the service resources of different health agencies into one unit with government commitments and ongoing policy and financial supports. Furthermore, integrated services encourage health agencies to work together in a closely coordinated way, and emphasize a coordination mechanism for inter-secteral cooperation and collaboration [6]. We recommend the following strategies for improving coordination of the integrated service delivery:

1. Establish regular meetings between different health agencies to exchange information and address problems.
2. Establish a client referral scheme between different health agencies with uniform guidelines, clear roles and consistent evaluation.

However, this study has several limitations. Firstly, integrating vertical PHIV services with other health service is still in the exploratory stage in China. It was just in 2009 that integration of PMTCT services was extended to an integrated PMTCT policy that also includes preventing syphilis and hepatitis B transmitted from mother to child. The study may not have been able to tap the depths of the analysis as much as it wished to. Secondly, in the literature there are no criteria to evaluate coordination among the complex health systems, especially to measure coordination using quantitative data. This study has presented the results of coordination assessment using the data from interviews and routine monitoring system. The ideas from the interviewees may be only individual opinions rather general attitudes. The data from the monitoring system may be insufficient evaluate the effectiveness of integration of the services. Finally, only one city and one county were selected to do the fieldwork in this study. This sample size is rather small numerically to represent the effectiveness of delivery of integrated PMTCT services in whole Province. Thus, there is a need for further studies in the future.

## Conclusion

The key obstacles to the coordination for the delivery of integrated PHSHT services have been identified by the stakeholders’ needs assessment in this study. These challenges including the poorly integrated service resources and a lack of a mechanism for the coordinated management hindered the coordination of the delivery of these integrated services. These findings suggest that concrete operational strategies are needed to overcome these obstacles in order to improve coordination for the effective delivery of integrated services in China.

## Abbreviations

MTCT: Mother-To-Child Transmission
PMTCT: Prevention of Mother-To-Child Transmission of HIV
PHC: Primary Health Care
PHSHT: Prenatal HIV, Syphilis and Hepatitis B Testing
MCH: Maternal and Children Health care
CDC: Centre for Disease Control
IDT: Infectious Disease Treatment
CPSTD: Clinic for Prevention of Sexual Transmission Disease
